# *Drosophila PINK1* and *parkin* loss-of-function mutants display a range of non-motor Parkinson's disease phenotypes

**DOI:** 10.1016/j.nbd.2017.04.014

**Published:** 2017-08

**Authors:** Hannah Julienne, Edgar Buhl, David S. Leslie, James J.L. Hodge

**Affiliations:** a School of Physiology, Pharmacology and Neuroscience, University of Bristol, Biomedical Sciences Building, University Walk, Bristol BS8 1TD, United Kingdom; b Department of Mathematics and Statistics, Fylde College, Lancaster University, Lancaster LA1 4YF, United Kingdom

**Keywords:** Parkinson's disease, Non-motor symptoms, *PINK1*, *Parkin*, *Drosophila*, Learning, Memory, Circadian rhythms, Sleep, Electrophysiology, DD, Constant darkness, D/NI, Diurnal/nocturnal index, DA, Dopamine, DAM2, *Drosophila* Activity Monitor, l-LNv, Large ventral lateral neurons, LD, 12:12 h light-dark cycle, R_in_, Membrane input resistance, MCH, 4-methylcyclohexanol, PD, Parkinson's disease, PI, Performance Index, *PINK1*, PTEN-induced putative kinase 1, OCT, 3-octanol, RBD, REM sleep behaviour disorder, RMP, Resting Membrane Potential, RS, Rhythmicity statistic, SFR, Spontaneous firing rate, ZT, Zeitgeber time

## Abstract

Parkinson's disease (PD) is more commonly associated with its motor symptoms and the related degeneration of dopamine (DA) neurons. However, it is becoming increasingly clear that PD patients also display a wide range of non-motor symptoms, including memory deficits and disruptions of their sleep-wake cycles. These have a large impact on their quality of life, and often precede the onset of motor symptoms, but their etiology is poorly understood. The fruit fly *Drosophila* has already been successfully used to model PD, and has been used extensively to study relevant non-motor behaviours in other contexts, but little attention has yet been paid to modelling non-motor symptoms of PD in this genetically tractable organism. We examined memory performance and circadian rhythms in flies with loss-of-function mutations in two PD genes: *PINK1* and *parkin*. We found learning and memory abnormalities in both mutant genotypes, as well as a weakening of circadian rhythms that is underpinned by electrophysiological changes in clock neurons. Our study paves the way for further work that may help us understand the mechanisms underlying these neglected aspects of PD, thus identifying new targets for treatments to address these non-motor problems specifically and perhaps even to halt disease progression in its prodromal phase.

## Highlights

• *Drosophila PINK1* and *parkin* loss-of-function (LOF) mutants have memory deficits.
• *Drosophila PINK1* and *parkin* LOF mutants have weakened circadian rhythms.
• *Drosophila PINK1* and *parkin* LOF mutants have clock neuron electrophysiology defects.
• *Drosophila* is a powerful model for studying Parkinson's disease non-motor symptoms.

## 1 Introduction

Parkinson's disease (PD) is more commonly associated with its debilitating motor symptoms, which include tremor, rigidity and slowness of movement. These symptoms have been linked with the degeneration of dopamine (DA) neurons, and thus treatments for the disease have primarily been developed to treat symptoms by compensating for depleted levels of DA in the brain. However, it is becoming increasingly clear that PD patients also display a wide range of non-motor symptoms that most treatments are not specifically designed to address and may even make worse (Chaudhuri et al., 2006a, Chaudhuri et al., 2006b, Langston, 2006). These include problems related to cognition and disruption of the sleep-wake cycle. Cognitive impairments include memory problems and abnormalities related to reinforcement learning, in which DA is known to play an important role (Barone et al., 2011, Frank et al., 2004). Sleep impairments are particularly common, affecting up to two-thirds of PD patients, and include disorders such as insomnia, excessive daytime sleepiness and REM sleep behaviour disorder (RBD) (Barone et al., 2009, Mattis and Sehgal, 2016, Menza et al., 2010).

These aspects of the disease have typically attracted less attention than the hallmark motor symptoms, but there is growing interest in understanding how they arise, as they have a large impact on the quality of life of both patients and their carers, and their appearance can often precede the onset of motor symptoms (Barone et al., 2009). RBD in particular is thought to be a strong predictor of PD and dementia (Iranzo et al., 2013, Schenck et al., 2013). However, the etiology of non-motor symptoms is still poorly understood. It is not yet clear the extent to which they too result directly from the degeneration of DA neurons, as opposed to the dysfunction of other cell types. Matters are further complicated by possible adverse effects of medication and of the different symptoms on one another. For instance, symptoms of depression, which are commonly found in patients, may in turn cause sleep problems themselves, as can taking l-dopa medication at bed time. The benefits of using a simple, genetically tractable model organism in a controlled environment are clear in the face of such complications.

Although most cases of PD have no identifiable cause, some genetic mutations have been linked to familial cases of the disease, of which many affect genes that have homologs in the fly (Lu and Vogel, 2009). Here, we focus particularly on two genetic fly models of PD, with mutations in genes that are thought to act together in a mitochondrial quality control pathway: *PTEN-induced putative kinase 1* (*PINK1*) and the E3 ubiquitin ligase *parkin*. It is thought that *PINK1* accumulates on the outer membrane of damaged mitochondria, where it activates *parkin*, leading to the ubiquitination of *parkin* targets on the outer mitochondrial membrane. This ultimately results in the recruitment of autophagic machinery to degrade the defective mitochondria (von Stockum et al., 2016). It is thought that mitochondrial quality control may be particularly important in DA neurons, which are susceptible to oxidative stress (Subramaniam and Chesselet, 2013).

Loss-of-function mutations in *PINK1* and *parkin* in humans cause early-onset forms of PD (Kitada et al., 1998, Valente et al., 2004). Few studies have yet explored the impact of specific mutations on non-motor symptoms, but evidence suggests that patients with homozygous *parkin* mutations exhibit the usual range of PD sleep disorders (Limousin et al., 2009). *Drosophila PINK1* and *parkin* loss-of-function mutants exhibit a set of relevant phenotypes such as impaired locomotor activity, reduced longevity, mitochondrial abnormalities, and DA neuron degeneration (Greene et al., 2003, Park, 2006, Pesah et al., 2004, Whitworth et al., 2005, Yang et al., 2006). Neurophysiological studies are still in their infancy, but have detected abnormalities in synaptic signalling in larvae (West et al., 2015). Interestingly, rodent loss-of-function models have largely failed to replicate the hallmark symptoms of PD (Dawson et al., 2010).

*Drosophila* display many behaviours that are pertinent to modelling human disease, which are underlied by simple, tractable neural circuits. Learning and memory has been extensively studied using an olfactory associative learning assay, and DA has been shown to play a crucial role, as it does in mammals (Malik and Hodge, 2014, Tully and Quinn, 1985, Waddell, 2010). The fly has also been central to ongoing chronobiology research. Wild type flies are diurnal and show robust circadian rhythms in their activity in the absence of external time cues, their locomotor activity thus providing a convenient output of their internal clock (Rosato and Kyriacou, 2006). These behavioural fluctuations appear to be partly underpinned by fluctuations in the electrophysiological properties of pacemaker neurons expressing the neuropeptide pigment dispersing factor (PDF), including the large ventral lateral neurons (l-LNvs) (Peschel and Helfrich-Förster, 2011). These thus represent defined neurons in the clock neural circuit that can be recorded from Buhl et al., 2016, Chen et al., 2015.

Despite these conserved behaviours, little attention has been paid to modelling non-motor symptoms of PD in *Drosophila*, except for two studies using flies expressing mutated form of the human PD-related gene **α*-synuclein* throughout their brains. These flies displayed short-term memory deficits after sleep deprivation, as well as abnormal sleep and circadian rhythms (Gajula Balija et al., 2011, Seugnet et al., 2009, Aug).

The relative simplicity of the fly brain and its genetic tractability, along with the existence of a number of quantitative assays to study fly behaviour, means there is great untapped potential for studying non-motor symptoms of PD in this model organism. We examined learning and memory performance and circadian rhythms in *parkin*-null and *PINK1*-null flies, seeking to determine if these could model some non-motor aspects of PD as well as the previously-documented motor defects and neurodegeneration. We also performed electrophysiological recordings of l-LNv clock neurons in control and mutant genotypes revealing novel mechanisms of action of these disease-causing genes.

## 2 Methods

### 2.1 Fly stocks

*Drosophila* were raised on cornmeal, molasses and agar medium under standard conditions. The wild type strain used was *CSw* ^−^, obtained from Dr Scott Waddell (University of Oxford). *park*^25^ and *PINK1*^*B*9^ null mutants, *PINK1*^*RV*^ revertant allele controls and *UAS-PINK1-RNAi* flies were all obtained from Dr. Alex Whitworth (University of Cambridge) (Greene et al., 2003, Park, 2006, Yang et al., 2006). *Timeless* (*tim*)-*GAL4* flies (stock 27) were obtained from Dr. Ralf Stanewsky (University of Münster) (Buhl et al., 2016, Chen et al., 2015).

### 2.2 Learning and memory experiments

To test learning and memory in flies, we used the olfactory-shock aversive conditioning protocol (Malik and Hodge, 2014, Tully and Quinn, 1985). Experiments were conducted at 25 °C and 70% humidity in dim red lighting conditions, using the T-maze apparatus. The odours used were 4-methylcyclohexanol (MCH) and 3-octanol (OCT), dissolved in 10 ml of mineral oil at concentrations of 1:500 and 1:250 respectively. The negative shock reinforcement used for conditioning consisted of 1.5 s pulses of 60 V electric shock, with 3.5 s pauses between shocks.

For training, groups of 30–50 flies were collected into a training tube containing a copper grid covering its inside surface. After an initial resting period of 90 s to acclimatise the flies, the first odour for conditioning was attached to the training tube and was drawn over the flies by a pump. For shock-paired odours, the electric shock was simultaneously administered through the copper grid. The flies were exposed to each odour for 1 min with a 30 s break of fresh air in between.

For memory tests, flies were kept in food vials before being reintroduced to the maze for testing. For testing, the flies were introduced into the central compartment of the T-maze. After a 90 s resting period they were transferred to a decision point from which they were allowed to move freely into the two arms of the maze, each with a different odour attached. They were given 2 min to make their decision, after which time the number of flies in each arm was counted.

After counting the number of flies making a correct decision (moving into the arm away from the shock-paired odour) and the number making a wrong decision, a performance index (PI) was calculated:

(1) $$\begin{array}{lll} \text{PI}= & \left(\text{number of correct flies}-\text{number of incorrect flies}\right) & \\ & /\text{total number of flies}. \end{array}$$

A PI of 1 thus indicates 100% avoidance of the shock-paired odour (perfect learning) and a PI of 0 an even split (no learning). To eliminate any effects of odour bias, the assay was always performed with two groups of flies, one shocked with MCH and the other shocked with OCT. The average was then taken of the two scores to give n =1 PI value.

Control experiments were conducted to confirm that any decrements in PI scores were due to a central learning or memory deficit and not to a peripheral defect in odour acuity or shock reactivity. To test for odour acuity flies were given 2 min to decide between an odour at the concentration used for experiments and fresh air in the T-maze. The percentage of flies avoiding the odour was then recorded. Flies that can smell normally typically avoid odours and instead approach fresh air. To test for shock reactivity flies were given 2 min to decide between a tube administering an electric shock and a second identical tube that was not. The percentage of flies avoiding the shock was then recorded.

### 2.3 Circadian rhythm experiments

Locomotor activity was recorded using the TriKinetics *Drosophila* Activity Monitor (*DAM2*). In this system, flies are held individually in small horizontal glass tubes intersected by an infrared beam. When a fly is active it breaks this beam and activity is recorded (Chen et al., 2015, Rosato and Kyriacou, 2006, Schlichting et al., 2016). Here, we recorded activity of male flies on agar food in 30 min bins. Monitors were connected to the computer and placed in an incubator at 25 °C and 60–70% humidity. The flies were kept in LD (12:12 h light-dark cycle) for two full days before being switched to DD (constant darkness) for a further seven days. Three repeats of this experiment were conducted on separate occasions to ensure replicability.

Circadian rhythm analysis was performed in Matlab using the Flytoolbox (Levine et al., 2002b) with some modifications described below. Additional statistical analyses were done in GraphPad Prism. From the data recorded by the DAM system, double-plotted (each day is plotted twice) actograms were plotted to help visualise how the activity of the flies varies with time of day. By examining the actogram for an individual fly, it was classified as rhythmic if there was an obvious circadian rhythm to its activity, as arrhythmic if there was no such rhythm present, or as weakly rhythmic if there was only partial rhythmicity or if the rhythmicity changed over time.

The data from flies that died before the end of the experiment were excluded from analysis. The data from the remaining flies were then filtered using a low-pass Butterworth filter to eliminate any periodicities under 4 h. We used autocorrelation analysis to measure rhythmicity in DD, as has been done by others (Levine et al., 2002a). This method involves the cross-correlation of a signal with itself in time, which can then be plotted as an autocorrelogram. Significant periodic variation in this autocorrelogram indicates rhythmicity in the signal. The strength of any rhythmicity present was assessed by looking at the height of the third peak in the autocorrelogram, within a given range of periodicities (between 16 and 32 h in our case), and using this to calculate a rhythmicity statistic (RS). The RS is the ratio of the height of the third peak to the absolute value of the 95% confidence interval line. The autocorrelogram also gives an estimate of the period of any rhythmicity.

To complement this RS value, we also used a value termed the diurnal/nocturnal index (D/NI), which simply quantifies the distinction between day time and night time activity levels.

(2) D/NI=(day-time activity−night-time activity)/total activity.

This statistic has the benefit of being intuitive to calculate and independent of overall activity levels. It has previously been used by others to analyse the activity of flies in LD (Kumar et al., 2012). Here we have modified its use for analysing data in DD by designating the ‘day-time’ and ‘night-time’ periods for individual flies according to the period of their rhythmicity, as calculated by the autocorrelation analysis.

### 2.4 Electrophysiological recording of clock neurons

Whole-cell current clamp recordings were performed as described previously (Buhl et al., 2016, Chen et al., 2015, Schlichting et al., 2016). To visualise the l-LNvs, *Pdf::RFP* (Ruben et al., 2012) and a 555  nm LED light were used for control and experimental stocks. Adult male flies were collected either at Zeitgeber time (ZT) 1–3 (1–3  h after lights-on: day condition) or ZT13–15 (1–3  h after lights-off: night condition), where ZT0 corresponds to lights-on. For each genotype and time point brains from at least three different flies were used.

Whole fly brains were acutely dissected in extracellular saline solution containing (in mM): 101 NaCl, 1 CaCl_2_, 4 MgCl_2_, 3 KCl, 5 glucose, 1.25 NaH_2_PO_4_ and 20.7 NaHCO_3_ at pH  7.2. After removal of the photoreceptors, lamina, air sacks and trachea, a small incision was made over the position of the l-LNv neurons in order to give easier access for the recording electrodes. The brain was then placed ventral side up in the recording chamber, secured using a custom-made anchor and neurons were visualised using a  ×63 lens on an upright Zeiss microscope (Examiner.Z1, Carl Zeiss Microscopy GmbH, Jena, Germany). l-LNv neurons were identified on the basis of their fluorescence, size, morphology and position. Recordings were performed at room temperature (20–22 °C) using glass electrodes with 8–18  MΩ resistance filled with intracellular solution (in mM: 102 K-gluconate, 17 NaCl, 0.94 EGTA, 8.5 HEPES, 0.085 CaCl_2_, 1.7 MgCl_2_ or 4 MgATP and 0.5 NaGTP, pH  7.2) and an Axon MultiClamp 700B amplifier, digitised with an Axon DigiData 1440A (sampling rate: 20  kHz; filter: Bessel 10  kHz) and recorded using pClamp 10 acquisition software (Molecular Devices, Sunnyvale, CA, USA). Chemicals were acquired from Sigma (Poole, UK).

The liquid junction potential was calculated as 13  mV and subtracted from all the membrane voltages. A cell was included in the analysis if the access resistance was less than 50 MΩ. Resting membrane potential (RMP) and the spontaneous firing rate (SFR) were measured after stabilising for 2–3 min. The membrane input resistance (R_in_) was calculated by injecting hyperpolarising current steps and measuring the resulting voltage change. Neuron excitability was measured by injecting a 500  ms long positive current pulse with increasing amplitude up to +40 pA and manually counting the resulting spikes. The statistical tests were performed using Prism (GraphPad Software Inc., La Jolla, CA, USA).

## 3 Results

### 3.1 *PINK1* null flies display a learning impairment, while *parkin* null flies display a slower rate of memory decay following acquisition

Learning (two-minute memory) and intermediate-term memory (two-hour memory) was measured in *CSw^-^* wild type and *PINK1^B9^* and *park^25^* mutant flies. Young flies were used for all experiments presented here: although PD is generally a progressive disorder, these particular mutations cause an early-onset form of the disease and non-motor symptoms can be present long before clinical diagnosis. Furthermore, reduced longevity and other defects present in the flies make it logistically difficult to study their behaviour at more advanced ages.

**Fig. 1:**
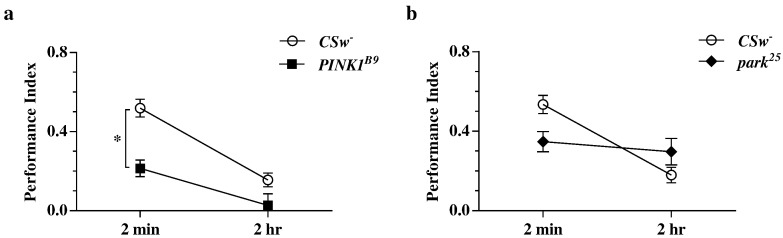
Two-minute and two-hour memory in *PINK1* and *parkin* loss-of-function mutants. (a) Comparing *PINK1^B9^* to wild type performance revealed a significant effect of both genotype (p = 0.0012) and time (p = 0.0011). (b) Comparing *park^25^* to wildtype revealed a significant effect of interaction between time and genotype (p = 0.0057) – while wild type flies display a significant difference between two-minute and two-hour memory (p < 0.01), this was not the case for *park^25^* flies. Data were analysed using two-way ANOVA with matching by day of experiment and Bonferroni's multiple comparisons tests (n = 7 experiments for each data point; * p < 0.05; error bars indicate standard error of the mean (SEM)).

*PINK1^B9^* flies have significantly lower PI scores compared with wild type, and display a significant impairment in two-minute memory in particular (Fig. 1a), which suggests a problem with memory acquisition rather than memory retention. Two hours after training, their PI scores drop to close to 0. Shock reactivity in these flies is normal and they significantly avoid both MCH and OCT, showing that they can smell both odours at the concentrations relevant for these experiments (Supplemental Fig. S1). Thus the lowered PI scores can be assumed to result from some central learning deficit as opposed to a peripheral sensory deficit.

Unlike *PINK1^B9^* flies, *park^25^* flies do not show a straightforward learning impairment, but rather an altered rate of decay of memory following acquisition – while wild type flies show a clear, significant decrease in PI scores over 2 h, the decrease in *parkin* mutants is small and not statistically significant (Fig. 1b). Although odour acuity is normal, mutant flies do display a small but significant reduction in shock reactivity compared with controls (Supplemental Fig. S1), which may explain the apparent (but not statistically significant) reduction in PI scores for two minute memory. However, this cannot clearly account for a slowing of memory decay and an apparent enhancement of PI scores for two hour memory. An additional experiment examining three-hour memory in *parkin* null mutants and both wild type and heterozygous controls further confirmed this trend of slower memory decay in the mutant flies (Supplemental Fig. S2). The fact that heterozygous controls performed similarly to wild type also means that any phenotypes seen in the mutants are highly unlikely to be due to some other background mutation.

### 3.2 Both *PINK1* and *parkin* null flies display weakened circadian rhythms in locomotor activity

We monitored the locomotor activity of 1–3  day old wild type and *PINK1^B9^* and *park^25^* mutant flies to see if they displayed normal circadian rhythmicity in their cycles of activity and rest in the absence of external time cues.

**Fig. 2:**
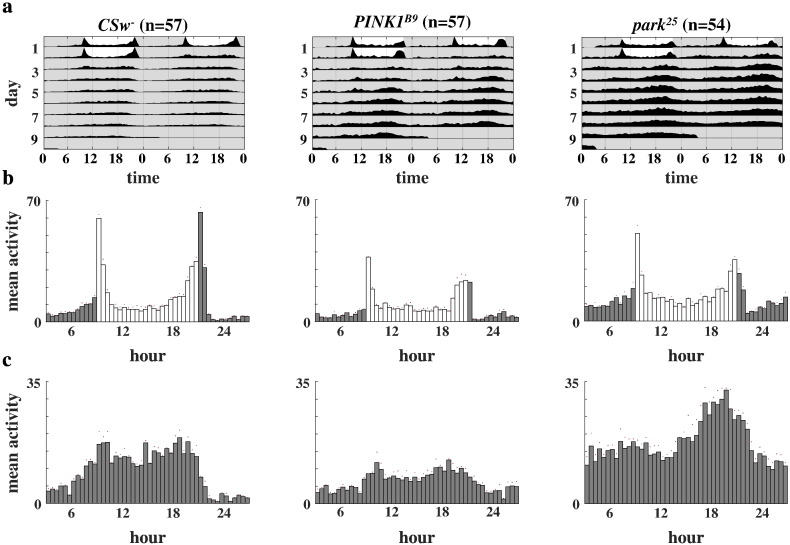
Averaged activity patterns of wild type and *PINK1* and *parkin* loss-of-function mutant flies in LD and DD conditions. The data were pooled from three separate rounds of recording. (a) Averaged double-plotted actograms of wild type and mutant flies over two days of LD and seven days of DD. (b) Averaged activity histograms for two days of LD conditions. (c) Averaged activity histograms for the first two days of DD conditions. Activity was measured in beam crosses per hour. The grey shading indicates times when lights were turned off. The red dots indicate SEM.

Wild type flies show characteristic morning and evening peaks in activity in LD, and maintain a rhythm of daytime activity and night-time inactivity in DD, with a period of just under 24 h (as illustrated by the leftwards trend in the actogram) (Fig. 2). The mutant genotypes display relatively normal activity in LD, with a slight reduction in the morning and evening peaks that can be attributed to their impaired locomotor activity (Fig. 2b). The *park^25^* flies also appear to have slightly elevated baseline activity levels, particularly at night. Both mutant genotypes do maintain some discernible rhythmicity in their activity in DD (Fig. 2a and c). However, there appears to be less of a distinction between the periods of activity and inactivity, and relative night-time activity seems to be elevated. *park^25^* flies also showed overall higher levels of activity.

**Fig. 3:**
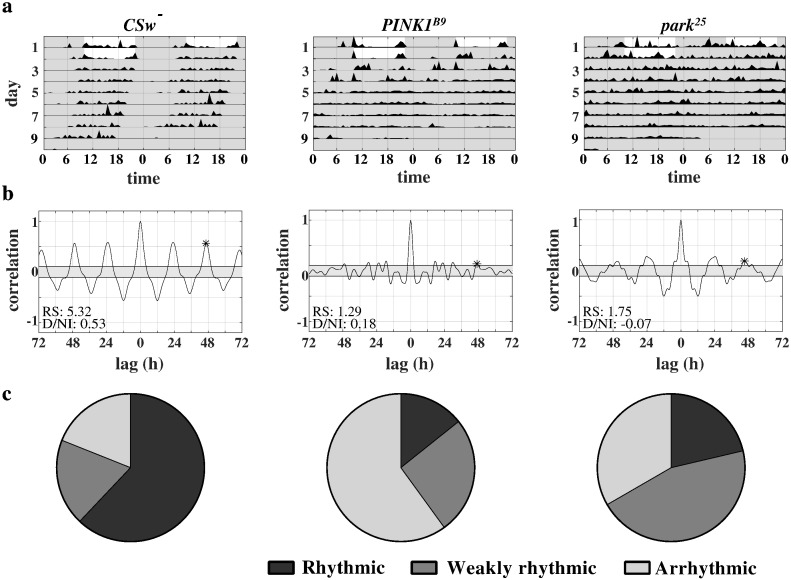
Activity patterns of individual flies in the absence of external time cues. (a) Representative double-plotted actograms of individual flies typical of each genotype over two days of LD and seven days of DD. (b) Autocorrelograms of the activity of these same flies in DD after a low-pass filter has been applied. The height of the third peak detected in the circadian range is highlighted, as this is the value used to calculate the rhythmicity statistic (RS) and period. The RS and diurnal/nocturnal index (D/NI) is listed for each fly. (c) Pie charts representing the overall proportion of flies classified as rhythmic, weakly rhythmic or arrhythmic in DD for each genotype.

Representative actograms for individual wild type and mutant flies can be seen in Fig. 3a and the corresponding autocorrelograms in Fig. 3b. Wild type flies, on the whole, show a robust rhythm of day-time activity and night-time inactivity in DD. *PINK1^B9^* flies are more likely to lack such a rhythm and *park^25^* flies tend to maintain some sort of circadian rhythm, but with less of a distinction between their day-time and night-time activity. The overall proportions of rhythmic, weakly rhythmic and arrhythmic flies for each genotype can be seen in Fig. 3c. While the majority of wild type flies displayed robust rhythmicity in DD, this was not the case for the mutant flies: most *PINK1^B9^* flies were totally arrhythmic, while more of the *park^25^* flies had a tendency to be weakly rhythmic.

**Fig. 4:**
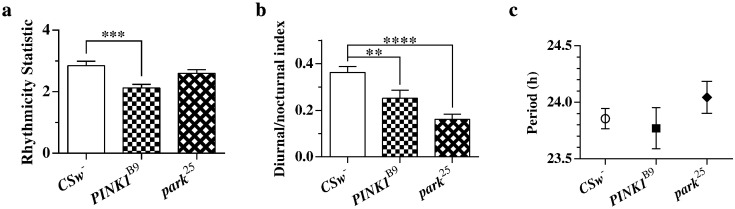
Analysis of circadian rhythms in wild type (n = 57), *park^25^* (n = 54) and *PINK1^B9^* (n = 57) mutants from three rounds of recording. (a) Rhythmicity statistic values for wild type and mutant flies. There was a significant difference among the means (p = 0.0004). (b) Diurnal/nocturnal index values for wild type and mutant flies. There was a significant difference between the means (p < 0.0001). (c) Period of rhythmicity for wild type (n = 52) and *park^25^* (n = 45) and *PINK1^B9^* (n = 36) mutants as calculated through autocorrelation analysis. Values obtained for arrhythmic flies were omitted. There was no significant difference detected between the means. Data were analysed using one way ANOVA with Bonferroni's multiple comparisons tests (**p < 0.01; ***p < 0.001; ****p < 0.0001; error bars indicate SEM).

*PINK1^B9^* mutants showed significant reductions in both RS and D/NI values compared with wild type, reflecting the reduced rhythmicity seen through observation (Fig. 4a and b). On the other hand, the *park^25^* flies did not show a significant reduction in RS values, but showed a large, highly-significant reduction in D/NI values. It seems that these flies are capable of maintaining some sort of circadian rhythm in DD, but that this does not manifest itself in the usual pattern of day-time activity and night-time rest. The period length of rhythms in both mutant genotypes was normal (Fig. 4c). Ageing did not affect the circadian rhythms of *PINK1* null mutants any more than it did those of wild type controls, further justifying the focus on young flies (Supplemental Fig. 3). Aged *parkin* null flies did not live long enough for experiments to be completed.

### 3.3 *PINK1* and *parkin* null flies show altered clock neuron electrophysiology

**Fig. 5:**
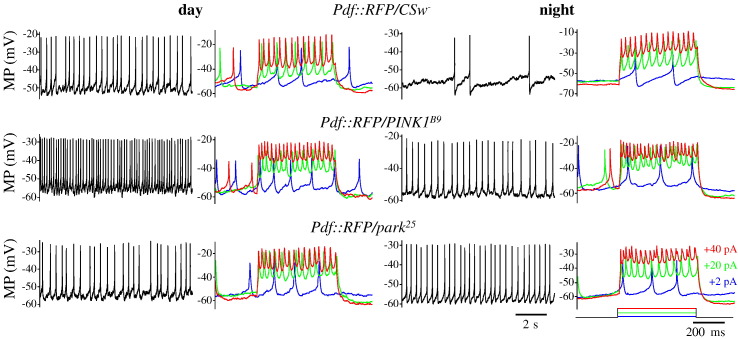
Electrophysiological characterisation of l-LNv clock neurons. MP: membrane potential. Spontaneous activity (left panels) and response to a current pulse (right panels, colour-coded as indicated) of wild type and mutant l-LNvs recorded at day (ZT1–3, left side) and night (ZT13–15, right side).

**Fig. 6:**
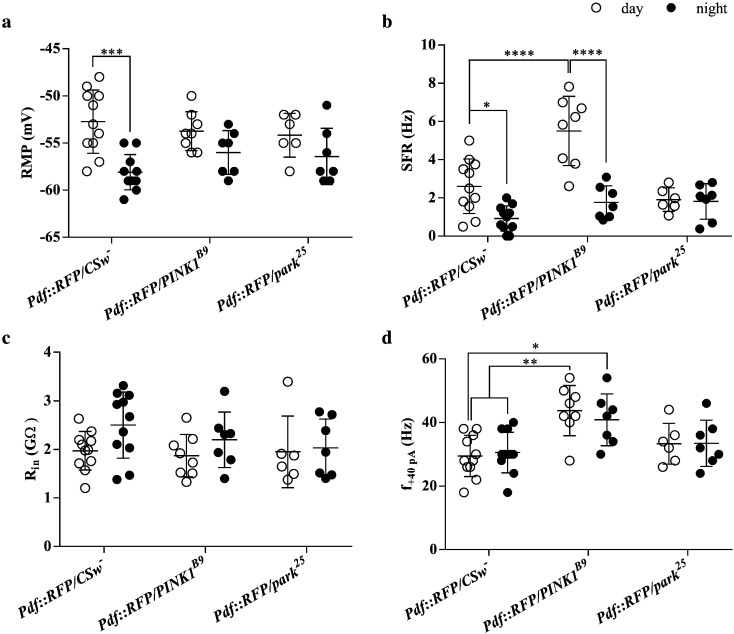
Quantitative analysis of electrophysiological properties of l-LNv clock neurons in wild type and mutant flies expressing *Pdf::RFP*, in both day and night conditions. (a) Analysis of resting membrane potential (RMP) values showed a significant effect of time of day (p < 0.0001) but no effect of genotype. (b) Analysis of spontaneous firing rate (SFR) values showed a significant effect of interaction between time of day and genotype (p < 0.001). *PINK1^B9^* neurons have a higher firing rate and *park^25^* neurons do not show a day/night difference. (c) Analysis of input resistance (R_in_) found a possible, but non-significant difference depending on time of day (p = 0.066) but no effect of genotype. (d) Analysis of responses to an injected current pulse (f_ +40 pA_) showed a significant effect of genotype (p ¡ 0.0001), with *PINK1^B9^* mutants being more excitable. Data were analysed using two-way ANOVA and Bonferroni's multiple comparisons tests (*p < 0.05; **p < 0.01; ***p < 0.001; ****p < 0.0001; error bars indicate standard deviations (SD)).

Since manipulation of PD genes led to impairments in circadian locomotor behaviour, we investigated potential underlying causes by recording from l-LNv clock neurons during the day and at night (Fig. 5) and measured electrophysiological properties of these cells (Fig. 6). As previously reported, wild type l-LNvs showed a more depolarised resting membrane potential (RMP) and a higher spontaneous firing rate (SFR; day/night ratio 2.83) in the day than at night, while input resistance (R_in_) and the response to an injected current pulse (+40  pA) did not differ significantly (Buhl et al., 2016).

l-LNv neurons in *PINK1^B9^* mutants also showed a day/night difference in SFR (day/night ratio 3.12) but with a higher firing frequency, especially in the daytime. The day/night difference in RMP was slightly less pronounced than in wild type. While R_in_ values were similar to wild type neurons, *PINK1^B9^* l-LNvs were more excitable and fired much more in response to injected current.

On the other hand, *park^25^* l-LNvs did not show a day/night difference in SFR (day/night ratio 1.05), and had a slightly reduced difference in RMP. R_in_ and current responses were similar to wild type neurons. This shows that mutations in *PINK1* and *parkin* genes differentially affect neurophysiological properties of clock neurons.

## 4 Discussion

We have shown that the established *PINK1* and *parkin* null *Drosophila* models of PD show changes in learning and memory and in circadian rhythms of locomotor activity, as well as underlying electrophysiological abnormalities in clock neurons. The persistence of these phenotypes when compared to additional heterozygous or revertant allele controls means they are unlikely to arise from other background mutations (Supplemental Figs. 2 & 4). This opens up the possibility of using these models to gain a better understanding of the mechanisms underlying non-motor symptoms of the disease, which are currently poorly understood, and are increasingly being highlighted as worthy of more detailed characterisation in animal models so that new targets for treatment may be identified (Dawson et al., 2010).

Our PD flies showed learning and memory abnormalities, correlating with the presence of cognitive impairments in patients (Barone et al., 2011, Frank et al., 2004). *PINK1* null flies showed lower memory scores compared with controls, and displayed a defect in memory acquisition in particular. This is not surprising considering the crucial role DA is known to play in reinforcement learning across species, in particular in providing the reinforcement signal or ‘reward prediction error’ (Schultz et al., 1997, Waddell, 2010). The results for *parkin* null flies did not show a simple impairment in the same way, but rather a slowing of memory decay. A possible explanation for this may come from recent research in *Drosophila* that implicates DA neurons in a forgetting process. According to this theory, ongoing tonic activity in a subset of DA neurons after learning induces the decay of memories, and inhibiting this activity boosts memory retention over time (Berry et al., 2012). It is conceivable that mutations in *parkin* might lead to dysfunction of these neurons, impairing their activity and thus slowing memory decay.

Although circadian rhythm defects are not usually explicitly cited as a feature of PD, up to 64% of patients report sleep problems and circadian processes are an important regulator of the sleep-wake cycle (Barone et al., 2009, Borb and Achermann, 1999). Disruptions to melatonin and cortisol regulation also provide evidence for a circadian component to sleep disturbances seen in the disease (Breen et al., 2014, Mattis and Sehgal, 2016, Videnovic et al., 2014). Furthermore, circadian disruptions have also been found in rodent PD models: mice overexpressing **α*-synuclein* show fragmented or reduced circadian locomotor activity accompanied by reduced firing of their clock neurons, while the MitoPark mouse shows a progressive loss of clock neurons accompanied by profound disruptions to locomotor rhythms under constant conditions (Fifel and Cooper, 2014, Kudo et al., 2011, Willison et al., 2013).

Our results showed defects in the circadian rhythms of locomotor activity in both *PINK1* and *parkin* null flies under constant darkness conditions. A large proportion of *PINK1* null flies were found to be totally arrhythmic, while the *parkin* null flies tended to show more of a weakening of rhythms, along with an overall increase in activity. Both mutant genotypes showed less of a distinction between periods of activity and inactivity, losing a recognisable day/night pattern. The relative increase in night-time activity in mutant flies is especially noteworthy considering the presence of locomotor defects – if anything, we would have expected that any problems with movement would lead to lower levels of activity. Although detailed sleep analysis was beyond the scope of this study, any increase in night-time activity naturally suggests a possible reduction in sleep, correlating with the symptoms of insomnia seen in humans. A reduction in nighttime sleep has also been observed in an **α*-synuclein* fly model of PD (Gajula Balija et al., 2011). Unlike this model, however, the PD models examined here did not show any alterations in period length, suggesting that the nature of the circadian defect seen may be underlied by slightly different mechanisms.

Our behavioural results are complemented by our detection of electrophysiological abnormalities in l-LNv clock neurons, which are thought to mediate arousal behaviour (Parisky et al., 2008). The hyperexcitability of these neurons in the *PINK1* null flies may be responsible for interrupting their sleep during the night period, thus disrupting their circadian rhythm. Indeed, others have found that hyperexcitation of these neurons does disrupt sleep (Sheeba et al., 2008). The lack of a day/night difference in firing rate in the *parkin* null flies is particularly interesting, as it mirrors the lack of such a distinction between their locomotor activity levels during the day and night periods.

It remains to be determined to what extent these phenotypes are due to DA deficiency as opposed to defects intrinsic to the clock neurons themselves. On the one hand, the presence of abnormalities in l-LNv clock neuron activity, in particular the hyperexcitability of those in *PINK1* null flies, does suggest the presence of some intrinsic changes in these neurons. Indeed, we have found evidence that knocking down *PINK1* in clock neurons using RNAi results in a weakening of circadian rhythms (Supplemental Fig. 5). Furthermore, the suggestion that lowered DA levels alone might lead to sleep disruption is somewhat at odds with the current theory of DA mediating wakefulness in flies (Kume et al., 2005, Ueno et al., 2012). On the other hand, the l-LNv clock neurons are only one part of a larger network mediating the sleep-wake cycle, and it is likely that the effects we see on spontaneous firing rates are in some part due to network effects. Interestingly, l-LNv neurons express DA receptors, and DA deficient flies have been shown to have weakened circadian rhythms (Hirsh et al., 2010, Shang et al., 2011). Furthermore, simply disrupting DA neuronal signalling in the absence of cell death has been shown to produce circadian activity phenotypes similar to those observed in the **α*-synuclein* model mentioned above (Gajula Balija et al., 2011). It is also worth noting that the period of behavioural rhythmicity in the PD flies examined here was not significantly altered – significant alterations in period length are a typical indication of molecular clock dysfunction. The results are, however, consistent with the disrupted clock neuron excitability affecting clock output.

Although PD is typically thought of as specifically affecting DA neurons, a whole range of other neurons and neurotransmitter systems are increasingly thought to be affected, as reflected in the widening variety of targets for emerging drug treatments (Barone, 2010, Brichta et al., 2013). For instance, degeneration of wake-active hypocretin neurons in the hypothalamus may result in sleep dysregulation (Fronczek et al., 2007, Thannickal et al., 2007). The extensive genetic toolbox available in the fly should thus prove useful for dissecting apart the roles and interactions of these different systems.

A remaining intriguing aspect of our results is the qualitative differences seen between the two mutant genotypes, considering *PINK1* and *parkin* are thought to act in the same pathway. We offer two potential explanations for this. Firstly, it may be the case that the pathology is simply more severe in *PINK1* null flies than in *parkin* null flies. Thus, with regard to memory performance, it may be the case that DA neurons in *parkin* null flies are impaired only to the point of reducing their ongoing tonic activity without impairing their ability to produce phasic activity sufficient to provide a reinforcement signal, while those in *PINK1* null flies are impaired to the point that phasic activity is also affected. This would highlight the need to take into account the subtleties of neuromodulation in neural circuits controlling behaviour, especially when developing treatments that alter neuromodulatory pathways. Indeed, some studies in humans show that reinforcement learning is impaired in PD patients both on and off their medication, but in different ways (Frank et al., 2004). The behavioural circadian rhythm defect in *PINK1* null flies might also be thought of as more severe, as a higher proportion of these mutants were arrhythmic than the *parkin* null flies. In humans, *PINK1* PD might be thought of as more severe than *parkin* PD to the extent that the latter does not typically present with Lewy body pathology (Kalinderi et al., 2016). The other possible explanation for the difference is that these two genes have independent roles outside of the mitochondrial quality control pathway in which they are most usually studied, and that this pathway is perhaps more complicated than previously thought. Indeed, our understanding of the function of the *PINK1*/*parkin* pathway in an *in vivo* setting is still somewhat lacking, and different mitochondrial phenotypes have been found under normal physiological conditions in flies compared with cultured cells (Devireddy et al., 2015, Grenier et al., 2013, Sung et al., 2016). Evidence for at least one diverging pathway involving *PINK1* independent from *parkin* comes from a study into the phenotypic effects of the fly homolog of *HtrA2*, which has been associated with an increased susceptibility to PD (Tain et al., 2009).

Crucially, non-motor symptoms are often present years before the onset of motor symptoms and the clinical diagnosis of PD in humans. This is especially true of sleep disturbances such as RBD: the majority of RBD sufferers will go on to develop PD or a related disorder (Schenck et al., 2013). Therefore, it is increasingly thought that sleep disorders such as RBD may represent a prodromal phase of PD that precedes clinical onset of the disease by on average 14  years (dos Santos et al., 2015, Iranzo et al., 2013). Thus non-motor disorders of PD may offer a presymptomatic window for study and treatment, allowing earlier intervention as well as the development of drugs that could actually target the causes of the disease as opposed to merely treating the symptoms when irreversible brain damage has already taken place. It has even been suggested that sleep and circadian regulation might be used as a therapeutic tool to treat the disease (Mattis and Sehgal, 2016, Videnovic et al., 2014).

## 5 Conclusions

The disruptions to non-motor behaviour we have detected in our PD flies is interesting on a number of fronts. Our study paves the way for further work that may help us understand the mechanisms underlying these neglected aspects of the disease and identify targets for new treatments to address them. Not only this, but in doing so, we may gain a greater understanding of the role of the neurotransmitters involved; for instance, the subtly different roles played by DA in learning and memory. Most excitingly, the fact that many of these symptoms arise much earlier than the onset of motor problems also gives rise to the possibility that studying them will bring more general insights into the etiology of the disease, potentially leading to the development of treatments that can halt disease progression entirely before irreversible neuronal loss occurs.

Finally, the fact that we have detected any non-motor dysfunctions at all is interesting in itself. The presence of such disruptions in fly models of the disease cannot be due to side effects of medication and is less likely to be linked to symptoms such as depression or external environmental factors. As such, it provides support for the idea that cognitive and circadian disruptions really are an intrinsic aspect of the disease.
