# Supplementary material for: Drosophila PINK1 and parkin loss-of-function mutants display a range of non-motor Parkinson's disease phenotypes
Source: Neurobiol Dis. 2017 Aug;104:15–23. doi: 10.1016/j.nbd.2017.04.014 (PMC5469398; doi:10.1016/j.nbd.2017.04.014)
Supplement: Supplementary file 1 — Supplementary material. [file mmc1.pdf]

## Supplemental material.

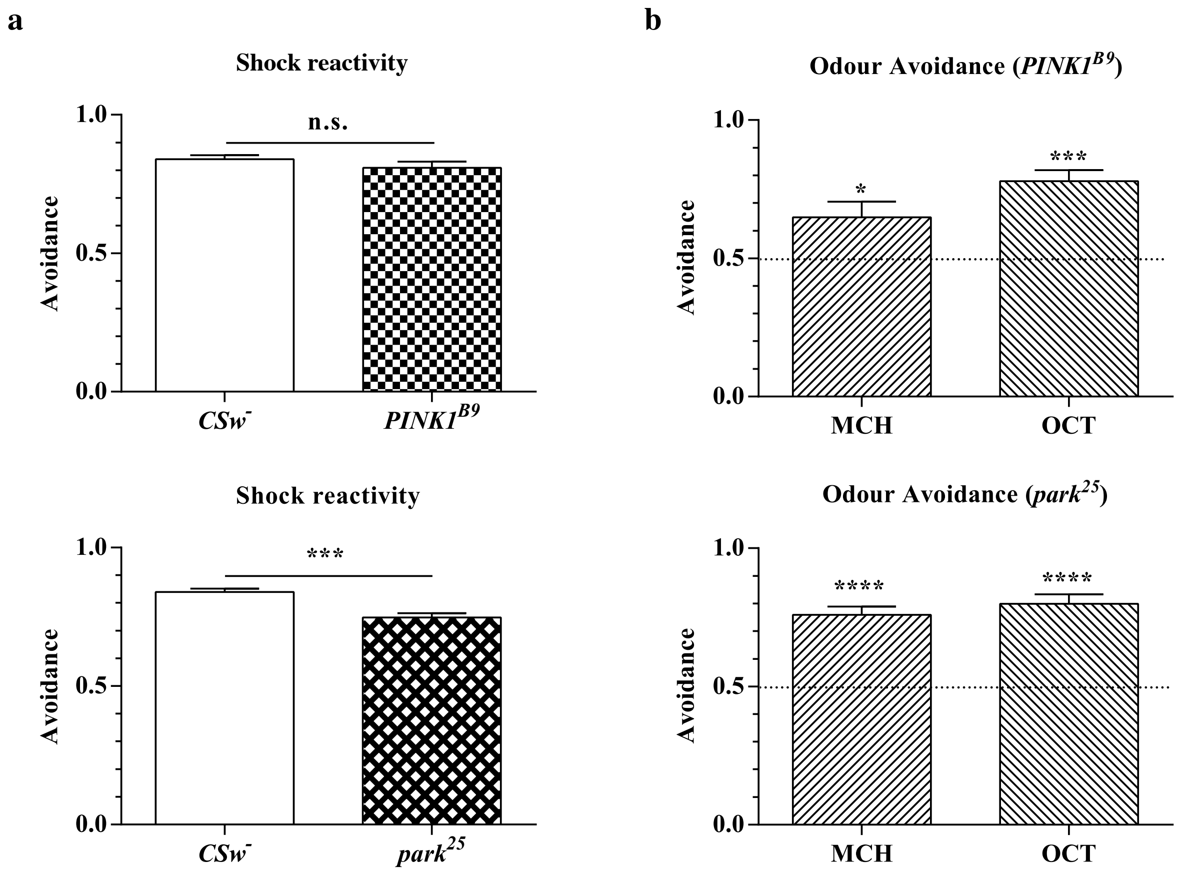


Figure S1: Controls for learning and memory experiments. **(a)** Shock avoidance scores for wild type and mutant flies. There was no significant difference in avoidance between wild type and *PINK1*$^{B9}$ flies (n=14) and a small but significant reduction in avoidance in *park*$^{25}$ flies (n=16). Data were analysed using a paired t-test. **(b)** Odour avoidance scores of mutant flies. Comparing mean avoidance with a score of 0.5 (corresponding to chance) showed that both mutant genotypes significantly avoided both MCH and OCT, indicating they can smell the odours. Data were analysed using a one-sample t-test (n=8; * $p<0.05$; *** $p<0.001$; **** $p<0.0001$; error bars indicate SEM).

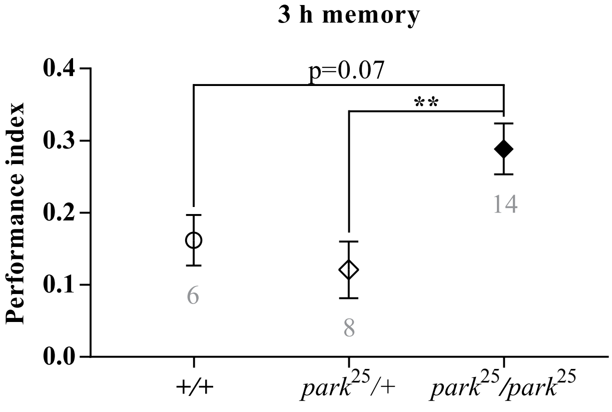


Figure S2: 3 h memory performance in *park*$^{25}$ mutants, and wild type and heterozygous controls. There was a significant difference between genotypes (p=0.0081). Data were analysed using one-way ANOVA and Bonferroni's multiple comparisons (n as shown; ** p<0.01; error bars indicate SEM).

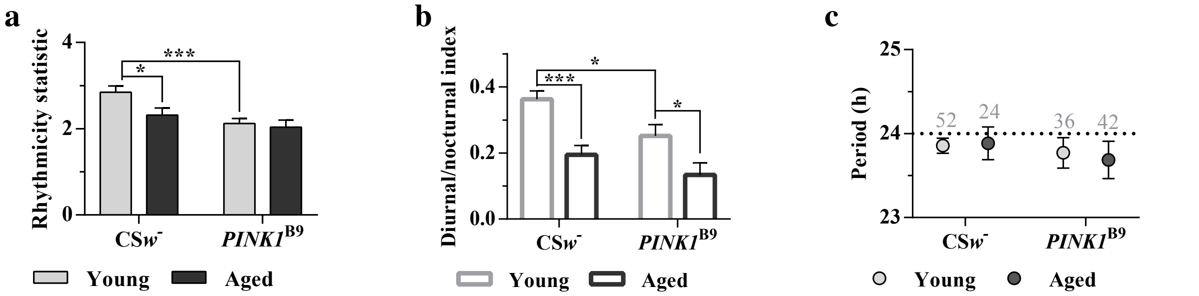


Figure S3: Analysis of circadian rhythms in young (1-3 days) and aged (8-14 days) CS*w*$^{-}$ wild type (n=57 young; n=34 aged) and *PINK1*$^{B9}$ mutants (n=57 young; n=42 aged). **(a)** RS values. There was a significant effect of both genotype (p=0.001) and age (p=0.0450). **(b)** D/NI values. There was a significant effect of both genotype (p=0.0089) and age (p<0.0001). **(c)** Period of rhythmicity for rhythmic and weakly rhythmic flies. There was no significant effect of either genotype (p=0.3934) or age (p=0.8711). Data were analysed using two-way ANOVA and Bonferroni's multiple comparisons. (* p<0.05; *** p<0.001; error bars indicate SEM)

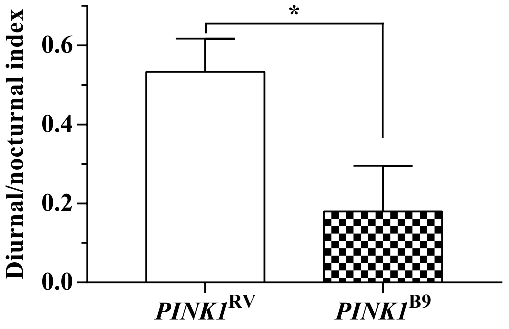


Figure S4: D/NI values for activity in DD of young (1-3 days) *PINK1*$^{RV}$ revertant allele controls (n=14) and *PINK1*$^{B9}$ mutants (n=8). Values were significantly lower in mutant flies (p=0.0209). Data were analysed using an unpaired t-test. (* p<0.05; error bars indicate SEM)

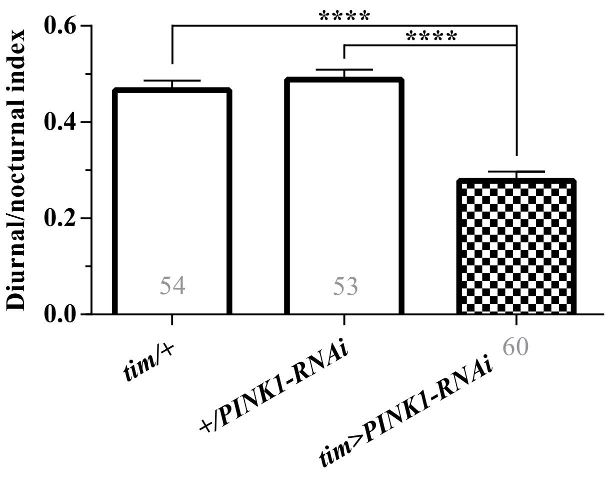


Figure S5: D/NI values for activity in DD of young (1-3 days) flies expressing *PINK1-RNAi* in clock neurons using the *tim-GAL4* driver. There was a significant difference between genotypes (p<0.0001). Data were analysed using Kruskal-Wallis tests and Dunn's multiple comparisons). (n as shown; **** p<0.0001; error bars indicate SEM; number in grey are *n*).
